# Extent of Implantoplasty in the Combined Surgical Therapy of Peri‐Implantitis: A Quasi‐Randomized Clinical Trial

**DOI:** 10.1111/cid.70144

**Published:** 2026-04-23

**Authors:** Alberto Monje, Ramon Pons, Conrado Aparicio, Dennis Tarnow, Paul S. Rosen, José Nart

**Affiliations:** ^1^ Department of Periodontology Universitat Internacional de Catalunya Barcelona Spain; ^2^ Department of Periodontology CICOM MONJE Institute Badajoz Spain; ^3^ Department of Periodontology ZMK University of Bern Bern Switzerland; ^4^ BOBI‐Bioinspired Oral Biomaterials and Interfaces, Department of Materials Science and Engineering, Barcelona East School of Engineering (EEBE) Universitat Politècnica de Catalunya‐BarcelonaTech (UPC) Barcelona Spain; ^5^ ICREA‐Catalan Institute for Research and Advanced Studies Barcelona Spain; ^6^ Department of Periodontics New York University College of Dentistry New York New York USA; ^7^ Department of Periodontics Rutgers University School of Dental Medicine, Newark New Jersey USA

**Keywords:** implant complications, implant disease, implant failure, implantoplasty, peri‐implant stability, peri‐implantitis

## Abstract

**Objective:**

The present study aimed to compare the clinical and radiographic performance of full‐length implantoplasty (FLIP) versus partial‐length implantoplasty (PLIP) for the supracrestal component as part of combined surgical therapy for peri‐implantitis with a ≥ 3 mm depth at the intrabony component.

**Materials and Methods:**

A single‐center, prospective, randomized, controlled, two‐arm comparative study was conducted to evaluate the extent of implantoplasty—limited to the supracrestal component (PLIP) or extending to both supracrestal and intrabony components (FLIP)—in the combined surgical management of peri‐implantitis. Clinical and radiographic outcomes were assessed 1 year after surgery. Disease resolution was defined using a composite of clinical and radiographic criteria, although sample size was calculated for pocket depth reduction. Generalized estimating equations were applied to calculate unadjusted and adjusted odds ratios.

**Results:**

A total of 33 patients (*N*_implants_ = 40) completed the study. All evaluated clinical parameters in both groups showed statistically significant changes over the study period. A significant intergroup difference was observed for modified sulcus bleeding index (mSBI), favoring FLIP (*p* = 0.003). Marginal recession (MR) was significantly greater in the FLIP group compared with the PLIP group (*p* = 0.006) and was more pronounced in the posterior mandible (*p* = 0.002). No other clinical parameters differed significantly between groups. Regarding marginal bone level (MBL) gain, FLIP demonstrated a statistically significant advantage over PLIP in the adjusted model (*p* = 0.009). For the remaining radiographic variables, significant changes were observed at the 1‐year follow‐up assessment, but no significant intergroup differences were detected. Overall disease resolution was achieved in 77.5% of cases. The adjusted model showed no statistically significant difference between the tested groups (OR = 14; *p* = 0.13). Smoking was consistently associated with less favorable clinical and radiographic outcomes. No major postoperative complications were reported.

**Conclusion:**

Combined surgical therapy for peri‐implantitis, including implantoplasty and regeneration of the intrabony component, is effective in arresting disease progression and restoring peri‐implant health. Extending implantoplasty to the contained intrabony compartment appears to provide additional clinical and radiographic benefits. However, this advantage comes at the expense of increased mucosal recession, highlighting the need for careful case selection and patient counseling.

## 1 Introduction

Historically, peri‐implantitis‐related bone defects have been regarded as having a circumferential, well‐contained morphology [1]. However, only more recently has there been an acknowledgment that they demonstrate a more crater‐like defect configuration, where the buccal plate is commonly missing [2]. The reason for this resides with the baseline alveolar bone dimension [3], where there is insufficient critical buccal bone thickness [4] or where implant position [5, 6] in relation to the skeletal bony envelope obviates this. Moreover, it must be highlighted that a relevant proportion of peri‐implantitis diagnosed on a daily basis exhibits a combined defect configuration, where there is the presence of both intrabony and supracrestal components [2]. These morphological features may jeopardize the stability of the clot and, therefore, the achievement of stable host bone along with any new bone formation.

Originally, Schwarz et al. described the protocol for the management of combined defects, using implantoplasty for the implant area outside the bony housing and regeneration for the surface residing within the contained intrabony compartment [7]. Subsequently, the same group demonstrated in a 4‐year report on advanced peri‐implantitis that, regardless of the surface decontamination method for the implant area within the intrabony compartment, bleeding tended to reduce by ~78% from baseline, and clinical attachment level increased by ~1.4 mm [8]. The 7‐year follow‐up of the aforementioned study corroborated the stability of these outcomes [9]. Schwarz et al. demonstrated that the combined approach in conjunction with soft tissue volume augmentation procedures was effective in reducing bleeding on probing (~75%), probing depth (~2.5 mm) and in gaining clinical attachment (~2 mm) [10]. More recently, Monje et al. demonstrated congruent findings with those previously reported. In terms of implant survival, the rate yielded was 90% over ~24 months of follow‐up. Disease resolution was 74% when a “flexible” definition of success (bleeding on probing ≤ 2 sites, probing pocket depth < 6 mm, and stable bone levels) was applied [11]. In addition, the width of KM at the buccal aspect was found to be an indicator of therapeutic success [12]. Furthermore, from the clinical perspective, it must be taken into account that surgical therapy to manage peri‐implantitis often leads to soft tissue changes (i.e., mucosal recession). Therefore, patient‐reported expectations must be cautiously evaluated a priori to assign the therapeutic prognosis, in particular in the esthetic area [13].

**FIGURE 1:**
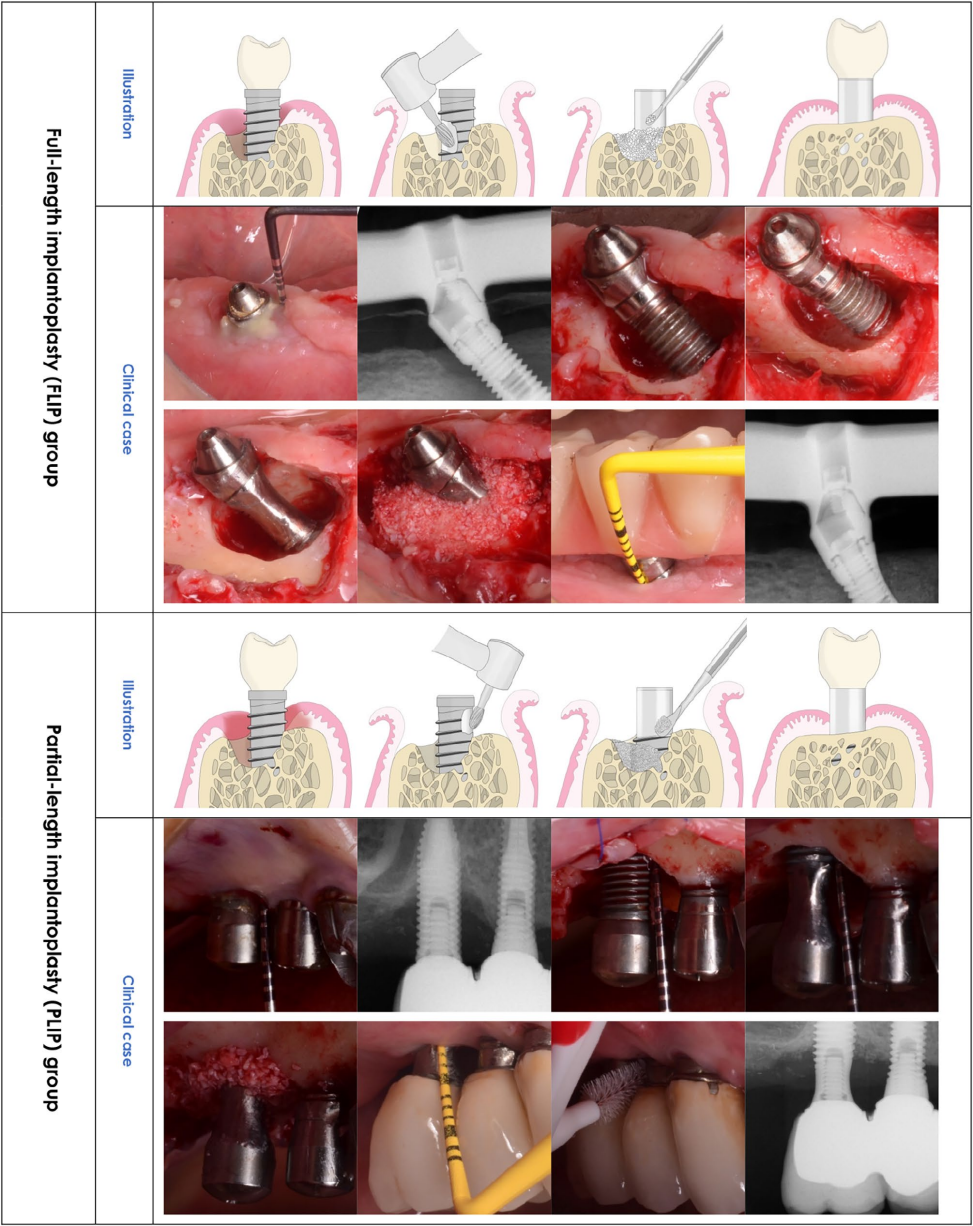
Clinical presentation and outcome for FLIP and PLIP.

One of the major challenges for success when managing peri‐implantitis intrabony components is surface decontamination. A myriad of agents and instruments have been described and tested in vitro and in vivo; yet, none has demonstrated consistent superiority. Hence, the dilemma is whether surface modification by implantoplasty can reduce implant surface roughness and effectively eliminate bacterial load and endotoxins, without jeopardizing the surface's positivity for enabling bone formation. With this in mind, the objective was to compare the clinical and radiographic performance of full‐length implantoplasty (FLIP) with partial‐length implantoplasty (PLIP) in the combined surgical management of peri‐implantitis (Figure 1).

## 2 Materials and Methods

**FIGURE 2:**
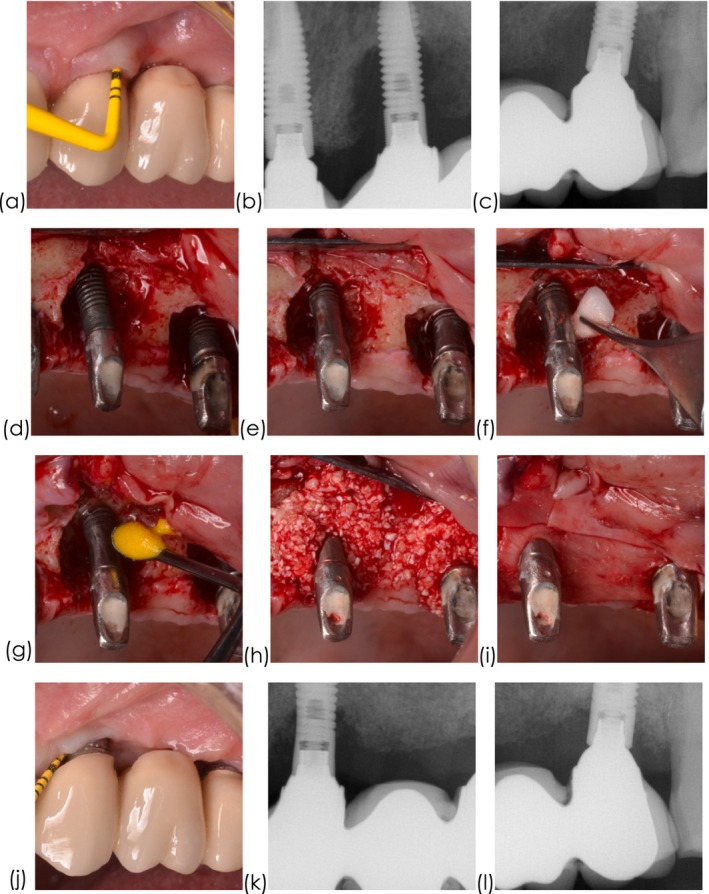
Representative case of FLIP. (a) clinical presentation following non‐surgical therapy, (b–c) periapical radiographic displaying advanced bone loss, (d) intra‐operative presentation showing combined defects, (e) implantoplasty along the supracrestal and intrabony components, (f) chemical decontamination applying hydrogen peroxide, (g) pharmacological decontamination using tetracycline chlorhydrate, (h) mixture of autogenous bone with xenograft, (i) collagen membrane is used to enhance graft stability, (j) clinical outcome showing disease resolution, (k–l) radiographic marginal bone level gain.

A single‐center, prospective, quasi‐randomized, controlled two‐arm comparative study was conducted in accordance with the Declaration of Helsinki on human studies, following approval from the Ethics Committee of the University of Extremadura (Badajoz, Spain). All patients received and signed a written informed consent form. Patient data was anonymized. The study was registered and approved at www.clinicaltrials.gov (NCT06131567) and is reported in accordance with the CONSORT guidelines [14]. This clinical trial was not registered before participant recruitment and randomization, as registration is not mandatory under local regulations before initiating patient enrollment (Figure 2).

### 2.1 Sample Size Calculation

The sample size was estimated according to the expected reduction in PPD (1 mm) and a standard deviation of 1.1 mm as reported elsewhere [15]. Accordingly, a sample size of 36 patients with a minimum of 44 implants was deemed suitable to identify a difference of 1 mm of pocket depth at final evaluation between the tested groups with a power of 80%, 95% confidence and considering a dropout of 10% of the sample and an intra‐class correlation of 0.2 [16, 17].

### 2.2 Study Sample

Consecutive patients diagnosed with peri‐implantitis exhibiting combined defects were recruited and evaluated from October 2021 to September 2025. The following inclusion criteria were applied: patients aged over 18, light smokers (< 10 cigarettes/day), former or non‐smokers, with no uncontrolled systemic diseases or medications known to affect bone metabolism, and partially or completely edentulous individuals without active periodontal disease. No restrictions were placed on the extent of baseline bone loss or the anatomical region. However, candidate implants for inclusion in this therapy shall be ≥ 3.5 mm in diameter. Only peri‐implantitis defects exhibiting type IB (with the implant outside the bony housing), IIIB, and IIIC were included [2]. Peri‐implantitis‐related bone defects had to display an intrabony component ≥ 3 mm in depth and a supracrestal compartment or an area of the implant outside the skeletal bony housing. Exclusion criteria included uncontained or shallow (< 3 mm) contained peri‐implantitis‐related bone defects where reconstructive therapy was not suitable, and sites with less than 2 mm of keratinized mucosa on the buccal aspect. Furthermore, cement‐retained prostheses were excluded in cases where patients declined to sign the informed consent acknowledging the risk of prosthesis fracture during its retrieval.

### 2.3 Clinical Assessment

Peri‐implantitis was defined according to the 2017 World Workshop on Periodontal and Peri‐Implant Diseases, characterized by probing pocket depths ≥ 6 mm and bone levels ≥ 3 mm apical to the most coronal portion of the intraosseous part of the implant, as determined from periapical radiographs [18]. The following clinical parameters and indices were assessed at baseline (4–6 weeks following non‐surgical therapy), and final (12 months) timepoints: pocket probing depth (PPD) recorded in millimeters using a plastic/metal North Carolina probe, applying an approximate probing force of 0.2 N, modified sulcular bleeding index (mSBI) scored as 0–3 according to the extensiveness and severity of bleeding on probing, mucosal recession (MR) was defined as the distance in millimeters from the implant–abutment connection as a steady mark and the mucosal margin, keratinized mucosa (KM) around the dental implants, measured from the free mucosal margin to the mucogingival junction at the mid‐buccal position, to the nearest millimeter, and suppuration grading index (SGI) scored as 0–3 according to the extensiveness and severity of suppuration on probing. Peri‐implant probing was systematically performed following prosthetic removal. Only in isolated cases [3] was proving recorded with the prosthesis placed due to the favorable emergence profile for vertical and accurate probing.

### 2.4 Radiographic Assessment

Periapical radiographs were obtained using the long‐cone paralleling technique, assisted by an intraoral radiographic positioning system to minimize projection errors and ensure reproducibility. Radiographic variables were recorded at baseline and at the final follow‐up examination by an examiner (RP) blinded to treatment allocation. The examiner was not involved in the clinical procedures and performed all radiographic measurements according to pre‐defined and standardized criteria. Radiographic calibration was performed using the known implant thread distance as reference, and measurements were performed using image analysis software (ImageJ 2.0.0‐rc‐69/1.52n; National Institutes of Health, Bethesda, MD, USA). Intra‐examiner reliability was assessed before study initiation, achieving a k‐value ≥ 0.85 on a representative sample (20% of the total sample size). The assessed radiographic parameters included: [1]

− Marginal bone level (MBL): The vertical distance (mm) from the implant shoulder to the first radiographically detectable bone‐to‐implant contact, measured at both mesial and distal sites.
− Intrabony depth (ID): The vertical distance (mm) from the bone crest to the first radiographically detectable bone‐to‐implant contact, measured at both mesial and distal sites.
− Intrabony defect angle (DA): The angle formed between a vertical reference line drawn along the external surface of the implant and a second line following the contour of the peri‐implant bone defect.

### 2.5 Outcomes

The primary outcome was disease resolution. Secondary outcomes were marginal bone level, clinical, and radiographic parameters at 12‐month follow‐up.

### 2.6 Definition of Disease Resolution

Disease resolution was achieved at the final evaluation if the following criteria were met: ≤ 1 spot of BOP, no suppuration, PPD ≤ 5 mm, no progressive bone loss beyond standard error (SE) ≥ 1 mm.

### 2.7 Therapeutic Modalities

Oral hygiene instructions were provided during the diagnostic phase. All eligible patients diagnosed with peri‐implantitis underwent non‐surgical therapy, performed by a single operator (AM), at least 5–6 weeks before the surgical reconstructive phase, as described elsewhere [19]. Implant prostheses were removed whenever access was not deemed suitable implantoplasty and healing abutments were placed. Regarding the surgical phase, a marginal internal bevel incision was performed to raise a full‐thickness flap, followed by debridement of granulomatous soft tissue using stainless steel curettes. Patients were then randomly assigned to either the test or control group, based on the last digit of their record number. The research assistant was designated to inform the operator once the flap was elevated and the granulomatous tissue was curetted. In test implants, FLIP (test group) was performed with tungsten carbide, followed by Arkansas and silicon burs until the level of roughness was deemed minimal. For the control group (PLIP), implantoplasty was performed only on the supracrestal component or the area outside the bony housing as described elsewhere using the same set of burs [20]. Following implantoplasty, the intrabony component of both groups was subjected to surface decontamination by means of NiTi brushes (Hans Korea Co., Gyeonggi‐do, Korea) at 600 rpm for approximately 2–3 min, hydrogen peroxide (3%) for 2 min, followed by tetracycline clorhydrate for 2 min and saline solution irrigation. Reconstructive therapy involved collecting autogenous bone chips using a back‐action chisel, which were subsequently combined in 1:1 ratio with anorganic cancellous bone (InterOss, SigmaGraft, Fullerton, USA). Platelet‐rich fibrin was centrifuged horizontally at 700RCF for 8 min in 10 mL tubes (Bio‐PRF, FL, USA). A membrane was used on top of the composite bone graft, with or without a collagen membrane, to increase graft containment as needed. Closure was achieved with 5.0 nylon sutures. All sites healed via a transmucosal (non‐submerged) healing approach. Patients were instructed to apply chlorhexidine 0.2% and chitosan gel 0.5% (5 mg/mg) to the treated area three times daily for 2 weeks, in conjunction with a 7‐day course of systemic amoxicillin (750 mg, two tablets per day). Additionally, an anti‐inflammatory regimen of ibuprofen (600 mg, one tablet every 5–6 h for 5 days) was prescribed. Sutures were removed within 2–3 weeks, after which the patients were advised to resume their standard oral hygiene practices. All patients adhered to a structured 4‐month recall program for supportive peri‐implant maintenance therapy. Following regular clinical evaluation, the use of air‐polisher devices using erythritol and ultrasonics was applied for periodontal and peri‐implant maintenance and any complications arising during the early healing phase were systematically documented and reported.

### 2.8 Statistical Analysis

Data were pseudonymized. Descriptive statistical analyses were conducted for continuous (mean, standard deviation [SD], median, quartiles) and categorical variables (absolute/relative frequencies). Simple binary logistic regression models were estimated using generalized estimating equations (GEE) to estimate the probability of disease resolution as a function of the independent variables. Unadjusted odds ratios (ORs) and 95% confidence intervals were obtained from the Wald chi‐square statistic test. Different correlation matrices were used to model the covariance structure (independence with robust estimators, interchangeable and unstructured with model‐based estimators), selecting the one that provided the best fit using the QIC index. Significant (*p* < 0.05) and relevant (*p* < 0.1) factors were then subjected to a final multiple model (adjusted ORs). For the dependent variables representing changes in clinical and radiographic parameters, expressed as differences between *T*_1_‐*T*_0_ times, simple linear regression models were applied, using a GEE approach, adjusted for independent factors and covariates. In this case, estimates for the regression beta coefficients with 95% confidence intervals were obtained and subsequently adjusted in a multiple model. The GEE analysis was justified by the within‐subject correlation inherent in the multi‐level structure of the data (patients who contributed several implants to the study). The significance level used in the analyses was 5% (α = 0.05). Tables S1–S9 provide the non‐adjusted and adjusted ORs and 95% confidence intervals for each of the clinical and radiographic variables evaluated.

## 3 Results

Patients were recruited and evaluated from October 2021 to September 2025. Overall, 36 patients were recruited. Three dropouts were reported due to incidents that precluded their attendance at the prescribed supportive peri‐implant therapy. Accordingly, 33 patients with 40 implants were included in the study. Of these subjects, 24 were women (72.7%) and 9 men (27.3%), with an overall mean age of 67.8 ± 10.9 years (range 31–90). Hence, the distribution of the groups was as follows: FLIP group: n_patients_ = 18 n_implants_ = 19 and PLIP group: n_patients_ = 15 n_implants_ = 21 (Figure S1). In total, 29 were non‐smokers, 7 were former smokers, and 4 were light smokers. Concerning periodontitis stage and grade, 20 were stage III, 2 were stage IV and the remaining were edentulous, while 1 was grade A, 21 was grade B, and 1 was grade C. All had received periodontal treatment in the past (or before the surgical intervention) and were deemed to be well controlled following re‐evaluation. The most common type of prosthesis was fixed partial dentures [21] followed by PF2, PF1, FP3, and SC. Regarding surface topographic features, 27 patients carried TiUnite (Nobel Biocare AB, Göteborg, Sweden), 4 TiOblast (Astra Tech, Mölndal, Sweden), 2 Osseotite (Biomet 3i LLC, Palm Beach, FL, USA). Implant positions were 57.5% posterior maxilla, 35% in the posterior mandible, 5% in the anterior maxilla, and 2.5% in the anterior mandible. Defect IIIB^2^ (52.5%) was the most common configuration, followed by IB^2^ (30.3%). The vast majority of defects were ≥ 50% in extension relative to implant length (65%), followed by moderate bone loss (30%), and only 5% were mild (< 25% in extension) peri‐implantitis defects. Mean MBL at baseline was 5.78 ± 1.39 mm, with a mean ID depth of 3.99 ± 1.56 mm. Only 5 cases (3 control and 2 test) required a barrier membrane due to the graft's poor stability. Homogeneity of both groups for the baseline demographic, radiographic, and clinical data according to the results of the Chi‐square test, the two‐sample t‐test, and the Mann–Whitney test *p*‐value was observed, except for age (*p* = 0.03) (Table 1).

**TABLE 1:** Demographical, clinical, and radiographical characteristics of implants included in the study: *N* (%), mean ± SD, *p*‐value from Chi^2^, Fisher's exact test, Mann–Whitney's test, and Wald's Chi^2^ test of GEE model at implant‐level.

	PLIP	FLIP	*p*
Patients (N)	15	18	
Gender			0.458 (Fis)
Male	3 (20%)	6 (33.3%)	
Female	12 (80%)	12 (66.7%)	
Age	63.9 ± 12.4	71.2 ± 8.3	0.030 (MW)
Smoking			0.731 (Chi^2^)
No	11 (73.3%)	14 (77.8%)	
Former	2 (13.3%)	3 (16.7%)	
Yes (light)	2 (13.3%)	1 (5.6%)	
Type of prosthesis			0.213 (Chi^2^)
FPD	11 (73.3%)	10 (55.6%)	
FP1	2 (13.3%)	1 (5.6%)	
FP2	1 (6.7%)	5 (27.8%)	
FP3	0 (0.0%)	2 (11.1%)	
SC	1 (6.7%)	0 (0.0%)	
Implant system			0.680 (Chi^2^)
Nobel Biocare	13 (86.7%)	14 (77.8%)	
AstraTech	1 (6.7%)	3 (16.7%)	
3i	1 (6.7%)	1 (5.6%)	
Stage			
III	13 (100%)	7 (77.8%)	
IV	0 (0%)	2 (22.2%)	
Grade			0.502 (Fis)
A	1 (7.1%)	0 (0%)	
B	12 (85.7%)	9 (100%)	
C	1 (7.1%)	0 (0%)	
Defect type			0.399 (Chi^2^)
IB	7 (46.7%)	3 (16.7%)	
IIIB	6 (40%)	10 (55.5%)	
IIIC	2 (13.3%)	5 (27.8%)	
Implants (N)			0.178 (MW)
1	10 (66.7%)	17 (94.4%)	
2	4 (26.7%)	1 (5.6%)	
3	1 (6.7%)	0 (0%)	
Implants (total)	21	19	
Location			0.666 (Chi^2^ W)
PM	14 (66.7%)	9 (47.4%)	
pm	6 (28.6%)	8 (42.1%)	
AM	1 (4.8%)	1 (5.3%)	
am	0 (0%)	1 (5.3%)	
PPD *T*_0_	7.02 ± 1.94	7.05 ± 1.39	0.944 (Chi^2^ W)
mSBI *T*_0_	1.87 ± 0.76	2.29 ± 0.89	0.160 (Chi^2^ W)
SGI *T*_0_	0.35 ± 0.39	0.55 ± 0.59	0.173 (Chi^2^ W)
MBL *T*_0_	−5.54 ± 1.04	−6.04 ± 1.68	0.223 (Chi^2^ W)
ID *T*_0_	3.76 ± 1.29	4.24 ± 1.81	0.331 (Chi^2^ W)
DA *T*_0_	41.9 ± 13.2	44.9 ± 12.6	0.473 (Chi^2^ W)
MR *T*_0_	0.62 ± 0.86	0.11 ± 0.46	0.168 (Chi^2^ W)
KM *T*_0_	2.90 ± 1.41	2.58 ± 1.17	0.441 (Chi^2^ W)
Defect extent			0.163 (Chi^2^ W)
AD	15 (71.4)	11 (57.9)	
MO	4 (19.0)	8 (42.1)	
MI	2 (9.5)	0 (0%)	
Early complications			0.160 (Chi^2^ W)
No	20 (95.2)	15 (78.9)	
Dehiscence	1 (4.8)	4 (21.1%)	

Abbreviations: AD, advanced; DA, defect angle; FP, full‐arch; FPD, fixed partial denture; ID, intrabony defect depth; KM, keratinized mucosa; MBL, marginal bone level; MI, mild; MR, mucosal recession; MO, moderate; mSBI, modified sulcular bleeding index; PPD, pocket probing depth; SC, single crown; SGI, suppuration grading index.

### 3.1 Clinical Outcomes

Mean PPD reduction from T_0_ to T_1_ was 3.83 ± 1.45 mm for FLIP and 3.53 ± 2.02 mm for PLIP, with statistical significance (*p* < 0.001) for both groups. No differences were noticed between the evaluated groups at T_1_ (*p* = 0.42). Smoking habit showed a statistically significant association with deeper residual PPD (*p* = 0.028). PPD reduction was 1.23 mm lower in the smoker cohort than in non‐smokers (*p* = 0.009). Former smokers also displayed with lower, but without reaching statistical significance, PPD reduction (*p* = 0.07). Baseline PPD was the strongest factor dictating PPD reduction (*p* < 0.001). With regard to mSBI, the mean reduction between T_0_ and T_1_ was 2.19 ± 0.88 mm for FLIP and 1.73 ± 0.74 mm for PLIP. Statistical significance was reached from T_0_ to T_1_ (*p* < 0.001). Likewise, a statistically significant difference was noticed between the evaluated groups (*p* = 0.003), favoring FLIP. Age (*p* = 0.04), baseline mSBI (*p* < 0.001), and implant system (*p* = 0.01) showed significance with mSBI reduction. For SGI, statistical significance was reached from T_0_ to T_1_ (*p* < 0.001). No difference was recorded in this parameter between the evaluated groups (*p* = 0.27). None of the tested parameters showed significance in the adjusted model. MR demonstrated a statistically significant increase (*p* = 0.02). Interestingly, MR was significantly higher for FLIP than for PLIP (*p* = 0.006) and in the posterior mandible (*p* = 0.002). KM decreased 0.68 ± 1.42 mm for FLIP and 0.95 ± 1.20 mm for PLIP, resulting in no significant differences (*p* = 0.55). The variables evaluated did not demonstrate statistical significance for this parameter (Table 2).

**TABLE 2:** Changes of clinical and radiographic parameters from *T*_0_ (baseline) to T_1_ (1‐year follow‐up). *p*‐value from Wald's Chi^2^ test of GEE model at implant‐level corrected by Bonferroni.

	PLIP			FLIP			Comparison
	*T*_0_	*T*_1_	*p*	*T*_0_	*T*_1_	*p*	*p*
PPD T_0_	7.02 ± 1.94	3.48 ± 1.02	< 0.001***	7.05 ± 1.39	3.22 ± 0.99	< 0.001***	0.42
mSBI T_0_	1.87 ± 0.76	0.14 ± 0.15	< 0.001***	2.29 ± 0.89	0.10 ± 0.13	< 0.001***	0.003
SGI T_0_	0.35 ± 0.39	0.02 ± 0.07	< 0.001***	0.55 ± 0.59	0.00 ± 0.00	< 0.001***	0.27
MR T_0_	0.62 ± 0.86	1.33 ± 1.24	0.002**	0.11 ± 0.46	1,84 ± 1.07	< 0.001***	0.006
KM T_0_	2.90 ± 1.41	1.95 ± 2.01	0.002**	2.58 ± 1.17	1.89 ± 0.99	0.058	0.38
MBL T_0_	5.54 ± 1.04	3.93 ± 1.51	< 0.001***	6.04 ± 1.68	3.62 ± 1.47	< 0.001***	0.009
ID T_0_	3.76 ± 1.29	1.74 ± 1.05	< 0.001***	4.24 ± 1.81	1.77 ± 1.23	< 0.001***	0.40
DA T_0_	41.9 ± 13.2	62.0 ± 11.3	< 0.001***	44.9 ± 12.6	66.0 ± 12.8	< 0.001***	0.83

Abbreviations: DA, defect angle; ID, intrabony defect depth; KM, keratinized mucosa; MBL, marginal bone level; MR, mucosal recession; mSBI, modified sulcular bleeding index; PPD, pocket probing depth; SGI, suppuration grading index.

** *p* < 0.01.

*** *p* < 0.001.

### 3.2 Radiographic Outcomes

**FIGURE 3:**
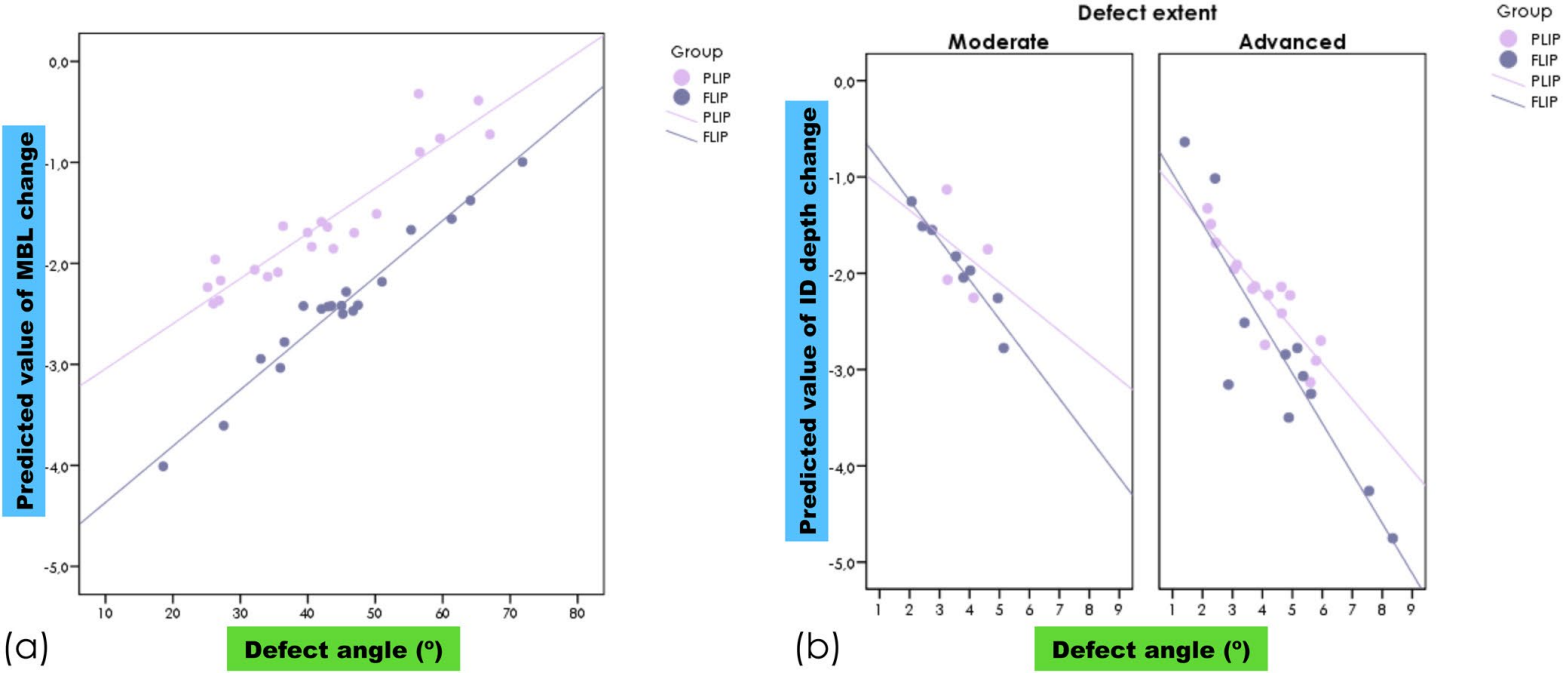
Plots showing the predicted value of marginal bone level change (a) and intrabony defect depth change (b) according to defect angle at baseline examination.

Marginal bone level increased 2.42 ± 1.20 mm for FLIP and 1.62 ± 1.27 mm for PLIP from T_0_ to T_1_. Variations were noticed during the study period (*p* < 0.002). Interestingly, FLIP outperformed PLIP in the adjusted model(*p* = 0.009). Concerning ID, a mean gain of 2.47 ± 1.36 mm for FLIP, compared with 2.02 ± 0.85 mm for PLPT, was achieved, with no statistically significant difference between the groups (*p* = 0.43). DA (*p* = 0.035) and baseline ID depth (*p* < 0.0001) were demonstrated to affect ID reduction. Regarding DA, an increase was recorded in both groups (FLIP = 21.1° ± 12.3°; PLIP = 20.1° ± 10.9°) (Figure 3). Increases in DA were observed during the study period (*p* < 0.001), but there were no significant differences between groups (*p* = 0.83).

### 3.3 Disease Resolution

**FIGURE 4:**
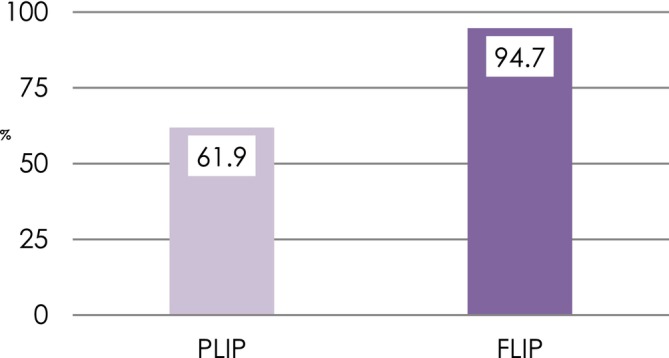
Disease resolution for PLIP and FLIP.

Overall, disease resolution was 77.5%. The disease resolution rate was 90.4% in the FLIP group and 64.6% in the PLIP group at T_1_. The non‐adjusted model supported the influence of the extent of implantoplasty at T_1_ (OR = 11.1; *p* = 0.04), favoring disease resolution for FLIP. However, the adjusted model did not yield statistical significance (OR = 14; *p* = 0.13) (Figure 4). Smoking was found to be the only variable that reached statistical significance (OR = 0.01; *p* = 0.013).

### 3.4 Postoperative Complications

Overall, 87.5% of the implant sites healed uneventfully, while 12.5% experienced early complications. Mucosal dehiscence during the early healing phase (< 21 days), affecting 12.5% of the implants was the reported complication without any sign of postoperative infection. No other early or late complications, including biomechanical complications, demanding interceptive therapy were reported during the study period.

## 4 Discussion

**FIGURE 5:**
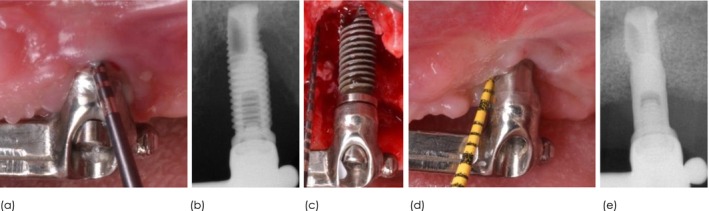
Representative case of FLIP. (a) clinical presentation following non‐surgical therapy, (b) advanced radiographic bone loss, (c) intraoperative image of the combined defect, (d) clinical disease resolution, (e) radiographic marginal bone level gain.

Implantoplasty was suggested as an adjunctive measure to minimize implant roughness and eliminate retentive features, such as implant threads, to enhance plaque control in the resective management of peri‐implantitis [21]. It was, later on, adopted in the surgical reconstructive therapy of defects exhibiting a combined supracrestal and intrabony component [7]. In this therapeutic modality, surface decontamination of the intrabony component was advocated by mechanical, chemical, electrolytic, and/or pharmacological means. The outcomes, however, in terms of complete removal of endotoxins and the organic component of the bacteria are suboptimal [22]. Moreover, surface topographic characteristics [23], the removal of the suprastructure [24] and bone defect morphology [25] have been demonstrated to impact the effectiveness of surface decontamination. More recently, subcrestal implantoplasty has been suggested as an effective alternative to decontaminate the implant surface while promoting reosseointegration, and facilitating oral hygiene around the implant which may account for the possibility that regenerative efforts might not be 100% successful [26]. We tested this hypothesis in a quasi‐randomized clinical trial. Interestingly, FLIP showed promise, as evidenced by clinical and radiographic outcomes. Despite not achieving statistical significance in the adjusted model, the likelihood of disease resolution was 14× higher with FLIP than with PLIP (Figure 5). This is the ultimate goal. While both in the short‐term demonstrated equivalent improvements, the fact that underlying disease/inflammatory issues remained leaves the implants susceptible to ongoing disease progression which is far from ideal. Moreover, based upon the suboptimal reconstructive outcomes (~2 mm) of the intrabony components, it is hypothesized that the risk for surface recontamination may increase. Hence, smoothing the implant surface may minimize bacterial biofilm contamination. Along these lines, mSBI and marginal bone level gain favored FLIP. On the other hand, FLIP showed greater MR than PLIP (Figure 2). Hence, findings from this study shed light on an alternative decontamination modality of the intrabony component of combined peri‐implantitis–related bone defects. It is worth highlighting that both treatment modalities succeeded with regard to disease resolution (77.5%) and halting progressive bone loss (95%) under complex scenarios, given the extension (65% were advanced) of the bony defects and the suboptimal containment. Despite the higher disease resolution rate demonstrated for FLIP, most of the clinical and radiographic parameters were found to be similar at the latest follow‐up. Hence, the reader shall interpret the findings with caution. Another major finding from this clinical trial was that smoking and baseline records are often associated to the inevitable outcome. These associations are not puzzling since similar outcomes have been noticed elsewhere applying other non‐surgical and surgical approaches to manage peri‐implantitis [27, 28, 29, 30].

Only a limited number of comparative clinical studies have explored the performance of implantoplasty as an adjunctive measure to resective or reconstructive approaches. Romeo et al. demonstrated that implantoplasty, in the resective therapy of peri‐implantitis, outperformed the control group in arresting disease progression and achieving peri‐implant health at 3‐year follow‐up [31, 32]. Lasserre et al., on the contrary, in a 6‐month follow‐up study did not yield differences between implantoplasty and glycine air abrasion in the non‐reconstructive therapy of peri‐implantitis. Schwarz et al., in a retrospective comparative clinical study, did not find an association between implantoplasty of the supracrestal component and reconstructing this zone in the short‐term following surface decontamination with titanium brushes [33]. More recently, two randomized clinical trials [34, 35] supported the inconsistency of implantoplasty on the resective therapy of peri‐implantitis, given the lack of added benefit in contrast to other less invasive surface decontamination methods. Our study is not comparable to those aforementioned since implantoplasty was used as an adjunctive measure aimed at both modifying and decontaminating the implant surface in the reconstructive therapy for peri‐implantitis. Previous non‐comparative clinical studies demonstrated the safety and effectiveness of implantoplasty when applied in the supracrestal component of combined defects [9, 11].

The successful reestablishment and long‐term maintenance of peri‐implant health following the delivery of treatment largely relies on effective plaque control by means of supportive care [36]. In this context, implantoplasty has demonstrated to result in a surface that is significantly less conducive to short‐term biofilm accumulation compared with a pristine implant surface with turned or modified surfaces [37]. Beyond its effects on biofilm accumulation, implant surface topography plays a critical role in modulating cellular behavior and tissue attachment. Its influence on connective tissue adhesion has been extensively documented on both pristine implant surfaces [38] and surfaces modified by implantoplasty [39, 40]. In this sense, previous studies have demonstrated that human gingival fibroblasts exhibit greater spreading on smooth surfaces than on rougher, indicating that connective tissue adhesion is strongly influenced by surface characteristics [41, 42, 43, 44]. Similarly, implant surface topography and morphology affect osteoblast growth, migration, and differentiation, as well as extracellular matrix synthesis and mineralization [45, 46]. In vitro studies have demonstrated that osteoblasts tend to adhere more rapidly to rougher titanium surfaces, although enhanced cell spreading and attachment have also been reported on machined surfaces [47, 48, 49]. Findings from this study could not assess the performance of fibroblasts or osteoblasts and their interaction with the modified implant surface following implantoplasty. Nevertheless, short‐term findings from this study suggested that FLIP/PLIP are safe procedures that promote disease arrestment comparable to other interventions to manage peri‐implantitis [50, 51, 52, 53].

Notably, cellular attachment may be influenced not only by surface topography but also by surface chemistry [54]. Supporting this concept, an in vitro study using Saos‐2 osteoblasts demonstrated that, although implantoplasty was the only peri‐implantitis treatment modality to significantly modify titanium surface morphology, composition, and wettability, it also promoted the expression of osteogenic markers such as alkaline phosphatase, osteoprotegerin, and osteocalcin, confirming its biocompatibility [55]. Conversely, an analysis of metal particle release from Ti‐6Al‐4 V implant surfaces during implantoplasty demonstrated that vanadium ion levels exceeded those of other metallic ions, and that cell viability assays showed that these particles caused a significant reduction in cytocompatibility for both osteoblasts and fibroblasts [56]. However, it is important to emphasize that, nowadays, most implant companies do not manufacture the Ti‐6Al‐4V alloy anymore. In fact, none of the implants included in the present study were titanium grade V. In old‐fashioned dental implants, therefore, cautiousness must be executed when planning implantoplasty due to potential vanadium toxicity. Additionally, the presence of titanium particles in the tissues surrounding dental implants is a common finding. Various mechanisms have been described as potential sources of titanium release, including friction during implant insertion, corrosion of the implant surface, micromovement and wear at the implant–abutment interface, and several techniques used for implant surface decontamination [57]. More recently, a clinical study in which soft‐tissue biopsies were collected from implant sites with and without peri‐implantitis reported that titanium microparticles are commonly detected in peri‐implant tissues and are not exclusively associated with the occurrence of peri‐implantitis [58]. However, although particles are present in both health and disease, in peri‐implantitis lesions, higher titanium particle density was associated with differential expression of immune‐related genes and increased RASGRP2‐positive cells, suggesting activation of NF‐κB–related inflammatory pathways. Hence, in light of the present findings, it has been debated whether titanium particles are clinically relevant.

From another perspective, implantoplasty inevitably reduces implant mass and resistance area, which may compromise implant strength and reduce fracture load, thereby increasing the risk of implant fracture. This risk appears to be higher in implants with platform diameters below 3.5 mm [59, 60] and may also be influenced by the type of implant connection [61]. In line with this, a laboratory study assessing whether the mechanical impact of implantoplasty depends on implant design, diameter, and material reported no clinically relevant reduction in strength in most cases; however, single narrow‐diameter titanium tissue‐level implants exhibited a potentially increased risk of mechanical complications following implantoplasty [62]. Moreover, the likelihood of mechanical complications—particularly fractures at the implant platform—seems to be associated with the extent of peri‐implant bone loss, as implants with more apically positioned bone levels appear more susceptible to fracture [63]. However, although numerous in vitro studies have demonstrated an increased risk of implant fracture when implants subjected to implantoplasty are exposed to cyclic loading under laboratory conditions [59, 62, 64], the available clinical evidence does not seem to support these findings [65]. In agreement with this data, no implant failure/fracture was reported in the present study. Certainly, only standard‐ or wide‐diameter implants and only 1 SC that completed the study. Hence, this could bias the biomechanical performance outcomes.

Several limitations of this study must be acknowledged. First, there is limited heterogeneity in implant surface topography. Modified surfaces have demonstrated a less favorable response to therapy than machined surfaces [66]. All the implants included in the present study were at the bone level and exhibited roughened surfaces. It might be speculated that tissue‐level implants or machined implants may not need surface modification strategies for effective decontamination and to minimize bacterial recolonization. It is also important to note that barrier membranes were used only when deemed necessary to enhance graft containment. Clinical evidence has consistently demonstrated that the use of membranes does not have an added benefit in the management of intrabony defects [67, 68]. Subset analysis did not yield significant differences in clinical and radiographic outcomes or postoperative complications. Moreover, 1‐year follow‐up is not sufficient to draw robust statements. Therefore, a longer follow‐up multicenter report of the included cases may elucidate the benefit of implantoplasty for the intrabony component of combined defects more consistently.

Importantly, if disease resolution was used for calculating the sample size with plausible event rates, the required sample would have been substantially larger. Therefore, this study should be viewed as an estimation study for that endpoint and future confirmatory trials should be powered specifically on disease resolution. With respect to clinical measurements, peri‐implant probing was systematically performed after prosthesis removal in the majority of cases; however, probing through the prosthesis in a limited number of implants may have introduced some degree of measurement error and should therefore be considered a minor limitation. Radiographic assessments were conducted by an independent examiner not involved in the surgical stage, using standardized protocols. However, complete blinding could not be fully ensured because radiographic changes in implant surfaces from implantoplasty could compromise blinding. This limitation should be considered when interpreting marginal bone level outcomes. Finally, caution is recommended when interpreting the findings, given that these approaches are intended for use only under specific site‐ and patient‐related features to achieve optimal outcomes. In addition, careful interpretation is warranted concerning the potential deleterious effects of titanium particles released during implantoplasty. Titanium particles have been reported to enhance osteoclast activation and may exert pro‐inflammatory effects by binding lipopolysaccharides and facilitating their intracellular uptake [69, 70]. This must be further explored in human clinical trials.

## 5 Conclusion

Combined surgical therapy for peri‐implantitis, including implantoplasty and regeneration of the intrabony component, is effective in arresting disease progression and restoring peri‐implant health. Extending implantoplasty to the contained intrabony compartment appears to provide additional clinical and radiographic benefits. Larger size and longer follow‐up studies are encouraged to validate this preliminary finding.

## Author Contributions

All the authors conceived the concept. A.M. performed the surgeries, R.P. and C.A. screened and analyzed the data. J.N. and P.S.R. supervised the writing.

## Funding

The present study was partially supported by SigmaGraft (Fullerton, CA, USA) and Scientific Foundation (Badajoz, Spain).

## Conflicts of Interest

The primary author receives honorarium and research funding for lecturing SigmaGraft (Fullerton, CA, USA), and owns royalties from the burs utilized in this study (Sanhigia, Zaragoza, Spain).

## Supporting information


**Figure S1:** CONSORT flowchart.

**Table S1:** Resolution by Group, patient's profile, and implant and defect characteristics: Results from simple and multiple binary logistic regression using GEE, non‐adjusted and adjusted OR, and 95% confidence intervals.
**Table S2:** Changes in MBL by Group, patient's profile, and implant and defect characteristics: Results from simple and multiple linear regression using GEE, non‐adjusted and adjusted beta coefficients, and 95% confidence intervals.
**Table S3:** Changes in PPD by Group, patient's profile, and implant and defect characteristics: Results from simple and multiple linear regression using GEE, non‐adjusted and adjusted beta coefficients, and 95% confidence intervals.
**Table S4:** Changes in mSBI by Group, patient's profile, and implant and defect characteristics: Results from simple and multiple linear regression using GEE, non‐adjusted and adjusted beta coefficients, and 95% confidence intervals.
**Table S5:** Changes in SGI by Group, patient's profile, and implant and defect characteristics: Results from simple and multiple linear regression using GEE, non‐adjusted and adjusted beta coefficients, and 95% confidence intervals.
**Table S6:** Changes in MR by Group, patient's profile, and implant and defect characteristics: Results from simple and multiple linear regression using GEE, non‐adjusted and adjusted beta coefficients, and 95% confidence intervals.
**Table S7:** Changes in KM by Group, patient's profile, and implant and defect characteristics: Results from simple and multiple linear regression using GEE, non‐adjusted and adjusted beta coefficients, and 95% confidence intervals.
**Table S8:** Changes in ID by Group, patient's profile, and implant and defect characteristics: Results from simple and multiple linear regression using GEE, non‐adjusted and adjusted beta coefficients, and 95% confidence intervals.
**Table S9:** Changes in DA by Group, patient's profile, and implant and defect characteristics: Results from simple and multiple linear regression using GEE, non‐adjusted and adjusted beta coefficients, and 95% confidence intervals.

## Data Availability

The data that support the findings of this study are available from the corresponding author upon reasonable request.
