# Supplementary material for: Extent of Implantoplasty in the Combined Surgical Therapy of Peri‐Implantitis: A Quasi‐Randomized Clinical Trial
Source: Clin Implant Dent Relat Res. 2026 Apr 23;28:e70144. doi: 10.1111/cid.70144 (PMC13107093; doi:10.1111/cid.70144)
Supplement: Supplementary file 1 — Figure S1: CONSORT flowchart. [file CID-28-0-s001.doc]

**Supplementary figure 1.** CONSORT flowchart

**Allocation**

**Analysis**

**Follow-Up**

**Enrollment**

Assessed for eligibility (n= 86)

Excluded (n= 40)

  Not meeting inclusion criteria (n= 34)

  Supported in referral practices (n= 6)

Analysed (n= 18)

Lost to follow-up (give reasons) (n= 18)

Allocated to intervention (n= 18)

 Received allocated intervention (n= 18)

Lost to follow-up (give reasons) (n= 15)

Not attending to maintenance (n= 3)

Allocated to intervention (n= 18)

 Received allocated intervention (n= 18)

Analysed (n= 15)

Randomized (n= 36)
