# Supplementary material for: Extent of Implantoplasty in the Combined Surgical Therapy of Peri‐Implantitis: A Quasi‐Randomized Clinical Trial
Source: Clin Implant Dent Relat Res. 2026 Apr 23;28:e70144. doi: 10.1111/cid.70144 (PMC13107093; doi:10.1111/cid.70144)
Supplement: Supplementary file 2 — Table S1: Resolution by Group, patient's profile, and implant and defect characteristics: Results from simple and multiple binary logistic regression using GEE, non‐adjusted and adjusted OR, and 95% confidence intervals. Table S2: Changes in MBL by Group, patient's profile, and implant and defect characteristics: Results from simple and multiple linear regression using GEE, non‐adjusted and adjusted beta coefficients, and 95% confidence intervals. Table S3: Changes in PPD by Group, patient's profile, and implant and defect characteristics: Results from simple and multiple linear regression using GEE, non‐adjusted and adjusted beta coefficients, and 95% confidence intervals. Table S4: Changes in mSBI by Group, patient's profile, and implant and defect characteristics: Results from simple and multiple linear regression using GEE, non‐adjusted and adjusted beta coefficients, and 95% confidence intervals. Table S5: Changes in SGI by Group, patient's profile, and implant and defect characteristics: Results from simple and multiple linear regression using GEE, non‐adjusted and adjusted beta coefficients, and 95% confidence intervals. Table S6: Changes in MR by Group, patient's profile, and implant and defect characteristics: Results from simple and multiple linear regression using GEE, non‐adjusted and adjusted beta coefficients, and 95% confidence intervals. Table S7: Changes in KM by Group, patient's profile, and implant and defect characteristics: Results from simple and multiple linear regression using GEE, non‐adjusted and adjusted beta coefficients, and 95% confidence intervals. Table S8: Changes in ID by Group, patient's profile, and implant and defect characteristics: Results from simple and multiple linear regression using GEE, non‐adjusted and adjusted beta coefficients, and 95% confidence intervals. Table S9: Changes in DA by Group, patient's profile, and implant and defect characteristics: Results from simple and multiple linear regression using GEE, non‐adjus [file CID-28-0-s002.docx]

**Supplementary table 1. Resolution by Group, patient´s profile and implant and defect characteristics:** Results from simple and multiple binary logistic regression using GEE, non-adjusted and adjusted OR and 95% confidence intervals.

|  | **Simple** | | |  | **multiple** | | |
| --- | --- | --- | --- | --- | --- | --- | --- |
|  | **OR** | **95% CI** | **p-value** |  | **OR** | **95% CI** | **p-value** |
| **Group** |  |  |  |  |  |  |  |
| PLIP | 1 |  |  |  | 1 |  |  |
| FLIP | 11.1 | 1.10 – 111.9 | **0.042** |  | 14.0 | 0.43 – 459.9 | 0.139 |
| **Gender** |  |  |  |  |  |  |  |
| Male | 1 |  |  |  |  |  |  |
| Female | 1.44 | 0.21 – 10.1 | 0.715 |  |  |  |  |
| **Age** | 1.04 | 0.97 – 1.11 | 0.264 |  | 0.99 | 0.91 – 1.08 | 0.829 |
| **Smoking** |  |  | **0.037** |  |  |  | **0.044** |
| No | 1 |  |  |  | 1 |  |  |
| Former | 0.15 | 0.01 – 1.84 | 0.140 |  | 0.13 | 0.01 – 1.80 | 0.127 |
| Yes | 0.04 | 0.01 – 0.56 | **0.018** |  | 0.03 | 0.01 – 0.48 | **0.013** |
| **Prosthesis** |  |  |  |  |  |  |  |
| FPD | 1 |  |  |  | 1 |  |  |
| Rest | 8.25 | 0.87 – 78.5 | **0.066** |  | 8.02 | 0.47 – 135.8 | 0.149 |
| **Implant system** |  |  | 0.631 |  |  |  |  |
| Nobel Biocare | 1 |  |  |  |  |  |  |
| AstraTech | 0.69 | 0.05 – 8.90 | 0.778 |  |  |  |  |
| 3i | 0.23 | 0.01 – 4.70 | 0.340 |  |  |  |  |
| **Location** |  |  | 0.802 |  |  |  |  |
| PM | 1 |  |  |  |  |  |  |
| pm | 1.29 | 0.17 – 9.73 | 0.802 |  |  |  |  |
| **Defect type** |  |  | 0.881 |  |  |  |  |
| IB | 1 |  |  |  |  |  |  |
| IIIB | 0.87 | 0.10 – 7.43 | 0.896 |  |  |  |  |
| IIIC | 1.67 | 0.11 – 24.7 | 0.710 |  |  |  |  |
| **Defect extension** |  |  |  |  |  |  |  |
| AD | 1 |  |  |  |  |  |  |
| MO | 4.89 | 0.60 – 40.0 | 0.139 |  |  |  |  |
| **Early complications** |  |  |  |  |  |  |  |
| No | 1 |  |  |  |  |  |  |
| Yes | 0.38 | 0.04 – 3.24 | 0.372 |  |  |  |  |
| **PPD T0** | 0.96 | 0.70 – 1.32 | 0.789 |  |  |  |  |
| **mSBI T0** | 0.90 | 0.35 – 2.32 | 0.821 |  |  |  |  |
| **SUP T0** | 1.71 | 0.20 – 14.3 | 0.620 |  |  |  |  |
| **MBL T0** | 0.93 | 0.60 – 1.44 | 0.735 |  |  |  |  |
| **Intrabony depth T0** | 0.92 | 0.60 – 1.41 | 0.696 |  |  |  |  |
| **Defect depth T0** | 1.02 | 0.96 – 1.09 | 0.502 |  |  |  |  |
| **Recession T0** | 0.85 | 0.36 – 2.02 | 0.719 |  |  |  |  |
| **KM T0** | 0.59 | 0.30 – 1.16 | 0.128 |  |  |  |  |

*p<0,05; **p<0,01; ***p<0,001

**Supplementary table 2. Changes in MBL by Group, patient´s profile and implant and defect characteristics:** Results from simple and multiple linear regression using GEE, non-adjusted and adjusted beta coefficients and 95% confidence intervals.

|  | **Simple** | | |  | **multiple** | | |
| --- | --- | --- | --- | --- | --- | --- | --- |
|  | **Beta** | **95% CI** | **p-value** |  | **Beta** | **95% CI** | **p-value** |
| **Group** |  |  |  |  |  |  |  |
| PLIP | 0 |  |  |  | 0 |  |  |
| FLIP | -0.80 | -1.58 -0.03 | **0.042** |  | -0.83 | -1.45 -0.20 | **0.009** |
| **Gender** |  |  |  |  |  |  |  |
| Male | 0 |  |  |  |  |  |  |
| Female | 0.29 | -0.95 1.52 | 0.651 |  |  |  |  |
| **Age** | -0.03 | -0.06 -0.01 | **0.039** |  | 0.01 | -0.05 0.05 | 0.887 |
| **Smoking** |  |  | 0.703 |  |  |  |  |
| No | 0 |  |  |  |  |  |  |
| Former | -0.33 | -1.71 1.06 | 0.643 |  |  |  |  |
| Yes | 0.69 | -1.33 2.71 | 0.501 |  |  |  |  |
| **Prosthesis** |  |  |  |  |  |  |  |
| FPD | 0 |  |  |  |  |  |  |
| Rest | -0.05 | -0.89 0.79 | 0.907 |  |  |  |  |
| **Implant system** |  |  | 0.121 |  |  |  |  |
| Nobel Biocare | 0 |  |  |  |  |  |  |
| AstraTech | -1.08 | -2.58 0.42 | 0.160 |  |  |  |  |
| 3i | 1.04 | -0.43 2.51 | 0.165 |  |  |  |  |
| **Location** |  |  |  |  |  |  |  |
| PM | 0 |  |  |  |  |  |  |
| pm | -0.42 | -1.37 0.54 | 0.391 |  |  |  |  |
| **Defect type** |  |  | 0.174 |  |  |  |  |
| IB | 0 |  |  |  |  |  |  |
| IIIB | 0.62 | -0.42 1.67 | 0.243 |  |  |  |  |
| IIIC | -0.42 | -1.82 0.98 | 0.555 |  |  |  |  |
| **Defect extension** |  |  |  |  |  |  |  |
| AD | 0 |  |  |  |  |  |  |
| MO | 0.20 | -0.55 0.96 | 0.595 |  |  |  |  |
| **Early complications** |  |  |  |  |  |  |  |
| No | 0 |  |  |  |  |  |  |
| Yes | 0.27 | -0.89 1.43 | 0.652 |  |  |  |  |
| **PPD T0** | 0.01 | -0.27 0.28 | 0.967 |  |  |  |  |
| **mSBI T0** | 0.23 | -0.31 0.76 | 0.403 |  |  |  |  |
| **SUP T0** | -0.56 | -1.34 0.23 | 0.166 |  |  |  |  |
| **MBL T0** | -0.36 | -0.61 -0.11 | **0.004** |  | -0.15 | -0.40 0.10 | 0.228 |
| **Intrabony depth T0** | -0.44 | -0.62 -0.26 | **<0.001** |  |  |  |  |
| **Defect depth T0** | 0.05 | 0.02 0.07 | **0.001** |  | 0.04 | 0.02 0.06 | **0.002** |
| **Recession T0** | 0.25 | -0.18 0.68 | 0.260 |  |  |  |  |
| **KM T0** | 0.23 | -0.02 0.48 | **0.069** |  | 0.13 | -0.17 0.43 | 0.405 |

**Supplementary table 3. Changes in PPD by Group, patient´s profile and implant and defect characteristics:** Results from simple and multiple linear regression using GEE, non-adjusted and adjusted beta coefficients and 95% confidence intervals.

|  | **Simple** | | |  | **multiple** | | |
| --- | --- | --- | --- | --- | --- | --- | --- |
|  | **Beta** | **95% CI** | **p-value** |  | **Beta** | **95% CI** | **p-value** |
| **Group** |  |  |  |  |  |  |  |
| PLIP | 0 |  |  |  | 0 |  |  |
| FLIP | -0.30 | -1.41 0.80 | 0.592 |  | -0.28 | -0.96 0.41 | 0.428 |
| **Gender** |  |  |  |  |  |  |  |
| Male | 0 |  |  |  | 0 |  |  |
| Female | -0.99 | -2.08 0.09 | 0.072 |  | -0.56 | -1.24 0.12 | 0.104 |
| **Age** | 0.04 | -0.01 0.09 | 0.101 |  | 0.01 | -0.02 0.03 | 0.542 |
| **Smoking** |  |  | **0.052** |  |  |  | **0.028** |
| No | 0 |  |  |  | 0 |  |  |
| Former | 0.82 | -0.09 1.74 | 0.077 |  | 0.57 | -0.07 1.21 | 0.078 |
| Yes | 1.23 | 0.22 2.25 | **0.017** |  | 0.77 | 0.19 1.36 | **0.009** |
| **Prosthesis** |  |  |  |  |  |  |  |
| FPD | 0 |  |  |  |  |  |  |
| Rest | -0.52 | -1.52 0.48 | 0.306 |  |  |  |  |
| **Implant system** |  |  | 0.204 |  |  |  |  |
| Nobel Biocare | 0 |  |  |  |  |  |  |
| AstraTech | 0.89 | -0.31 2.08 | 0.145 |  |  |  |  |
| 3i | -0.45 | -1.88 0.99 | 0.541 |  |  |  |  |
| **Location** |  |  |  |  |  |  |  |
| PM | 0 |  |  |  |  |  |  |
| pm | 0.57 | -0.59 1.73 | 0.335 |  |  |  |  |
| **Defect type** |  |  | 0.714 |  |  |  |  |
| IB | 0 |  |  |  |  |  |  |
| IIIB | -0.22 | -1.73 1.28 | 0.772 |  |  |  |  |
| IIIC | 0.39 | -1.39 2.17 | 0.669 |  |  |  |  |
| **Defect extension** |  |  |  |  |  |  |  |
| AD | 0 |  |  |  |  |  |  |
| MO | -0.36 | -1.76 1.04 | 0.613 |  |  |  |  |
| **Early complications** |  |  |  |  |  |  |  |
| No | 0 |  |  |  |  |  |  |
| Yes | -0.60 | -1.75 0.55 | 0.305 |  |  |  |  |
| **PPD T0** | -0.87 | -0.99 -0.74 | **<0.001** |  | -0.82 | -0.94 -0.71 | **<0.001** |
| **mSBI T0** | -0.39 | -1.04 0.26 | 0.236 |  |  |  |  |
| **SUP T0** | 0.09 | -0.68 0.86 | 0.815 |  |  |  |  |
| **MBL T0** | -0.24 | -0.54 0.06 | 0.122 |  |  |  |  |
| **Intrabony depth T0** | 0.16 | -0.14 0.47 | 0.284 |  |  |  |  |
| **Defect depth T0** | -0.01 | -0.05 0.03 | 0.643 |  |  |  |  |
| **Recession T0** | 0.32 | -0.34 0.97 | 0.343 |  |  |  |  |
| **KM T0** | -0.21 | -0.48 0.06 | 0.132 |  |  |  |  |

**Supplementary table 4. Changes in mSBI by Group, patient´s profile and implant and defect characteristics:** Results from simple and multiple linear regression using GEE, non-adjusted and adjusted beta coefficients and 95% confidence intervals.

|  | **Simple** | | |  | **multiple** | | |
| --- | --- | --- | --- | --- | --- | --- | --- |
|  | **Beta** | **95% CI** | **p-value** |  | **Beta** | **95% CI** | **p-value** |
| **Group** |  |  |  |  |  |  |  |
| PLIP | 0 |  |  |  | 0 |  |  |
| FLIP | -0.46 | -0.96 0.03 | **0.065** |  | -0.11 | -0.19 -0.04 | **0.003** |
| **Gender** |  |  |  |  |  |  |  |
| Male | 0 |  |  |  |  |  |  |
| Female | 0.03 | -0.57 0.63 | 0.929 |  |  |  |  |
| **Age** | 0.01 | -0.02 0.03 | 0.622 |  | 0.01 | 0.00 0.09 | **0.043** |
| **Smoking** |  |  | 0.621 |  |  |  |  |
| No | 0 |  |  |  |  |  |  |
| Former | 0.26 | -0.39 0.90 | 0.435 |  |  |  |  |
| Yes | -0.24 | -1.21 0.74 | 0.634 |  |  |  |  |
| **Prosthesis** |  |  |  |  |  |  |  |
| FPD | 0 |  |  |  |  |  |  |
| Rest | -0.02 | -0.58 0.54 | 0.952 |  |  |  |  |
| **Implant system** |  |  | **0.015** |  |  |  | **0.015** |
| Nobel Biocare | 0 |  |  |  | 0 |  |  |
| AstraTech | 0.73 | -0.11 1.56 | 0.087 |  | 0.18 | 0.05 0.32 | **0.008** |
| 3i | -0.44 | -0.88 0.01 | 0.053 |  | 0.09 | -0.09 0.28 | 0.317 |
| **Location** |  |  |  |  |  |  |  |
| PM | 0 |  |  |  |  |  |  |
| pm | -0.09 | -0.64 0.45 | 0.739 |  |  |  |  |
| **Defect type** |  |  | 0.251 |  |  |  |  |
| IB | 0 |  |  |  |  |  |  |
| IIIB | -0.05 | -0.70 0.61 | 0.890 |  |  |  |  |
| IIIC | -0.53 | -1.28 0.23 | 0.171 |  |  |  |  |
| **Defect extension** |  |  |  |  |  |  |  |
| AD | 0 |  |  |  |  |  |  |
| MO | -0.35 | -0.90 0.20 | 0.216 |  |  |  |  |
| **Early complications** |  |  |  |  |  |  |  |
| No | 0 |  |  |  | 0 |  |  |
| Yes | -0.51 | -0.85 -0.18 | **0.003** |  | 0.05 | -0.14 0.23 | 0.631 |
| **PPD T0** | -0.21 | -0.33 -0.08 | **0.002** |  | -0.01 | -0.03 0.02 | 0.615 |
| **mSBI T0** | -0.97 | -1.03 -0.92 | **<0.001** |  | -0.96 | -1.01 -0.90 | **<0.001** |
| **SUP T0** | 0.11 | -0.39 0.61 | 0.671 |  |  |  |  |
| **MBL T0** | 0.05 | -0.19 0.28 | 0.683 |  |  |  |  |
| **Intrabony depth T0** | 0.02 | -0.18 0.22 | 0.854 |  |  |  |  |
| **Defect depth T0** | -0.01 | -0.03 0.02 | 0.701 |  |  |  |  |
| **Recession T0** | 0.13 | -0.14 0.39 | 0.340 |  |  |  |  |
| **KM T0** | -0.14 | -0.27 -0.01 | **0.038** |  | 0.04 | -0.01 0.08 | 0.119 |

**Supplementary table 5. Changes in SGI by Group, patient´s profile and implant and defect characteristics:** Results from simple and multiple linear regression using GEE, non-adjusted and adjusted beta coefficients and 95% confidence intervals.

|  | **Simple** | | |  | **multiple** | | |
| --- | --- | --- | --- | --- | --- | --- | --- |
|  | **Beta** | **95% CI** | **p-value** |  | **Beta** | **95% CI** | **p-value** |
| **Group** |  |  |  |  |  |  |  |
| PLIP | 0 |  |  |  | 0 |  |  |
| FLIP | -0.22 | -0.54 0.10 | 0.184 |  | -0.02 | -0.06 0.02 | 0.279 |
| **Gender** |  |  |  |  |  |  |  |
| Male | 0 |  |  |  |  |  |  |
| Female | -0.04 | -0.43 0.35 | 0.843 |  |  |  |  |
| **Age** | -0.01 | -0.02 0.01 | 0.867 |  | 0.01 | -0.01 0.01 | 0.287 |
| **Smoking** |  |  | 0.544 |  |  |  |  |
| No | 0 |  |  |  |  |  |  |
| Former | -0.32 | -1.02 0.39 | 0.376 |  |  |  |  |
| Yes | 0.15 | -0.34 0.63 | 0.554 |  |  |  |  |
| **Prosthesis** |  |  |  |  |  |  |  |
| FPD | 0 |  |  |  |  |  |  |
| Rest | -0.28 | -0.64 0.07 | 0.117 |  |  |  |  |
| **Implant system** |  |  | **0.001** |  |  |  | 0.978 |
| Nobel Biocare | 0 |  |  |  | 0 |  |  |
| AstraTech | -0.17 | -0.83 0.50 | 0.622 |  | 0.00 | -0.01 0.01 | 0.960 |
| 3i | 0.38 | 0.16 0.59 | **0.001** |  | 0.01 | -0.02 0.02 | 0.834 |
| **Location** |  |  |  |  |  |  |  |
| PM | 0 |  |  |  |  |  |  |
| pm | -0.31 | -0.68 0.06 | 0.104 |  |  |  |  |
| **Defect type** |  |  | 0.260 |  |  |  |  |
| IB | 0 |  |  |  |  |  |  |
| IIIB | 0.29 | -0.09 0.66 | 0.132 |  |  |  |  |
| IIIC | 0.06 | -0.44 0.55 | 0.826 |  |  |  |  |
| **Defect extension** |  |  |  |  |  |  |  |
| AD | 0 |  |  |  |  |  |  |
| MO | 0.13 | -0.22 0.48 | 0.476 |  |  |  |  |
| **Early complications** |  |  |  |  |  |  |  |
| No | 0 |  |  |  |  |  |  |
| Yes | -0.07 | -0.61 0.46 | 0.793 |  |  |  |  |
| **PPD T0** | 0.01 | -0.06 0.08 | 0.812 |  |  |  |  |
| **mSBI T0** | 0.04 | -0.15 0.23 | 0.689 |  |  |  |  |
| **SUP T0** | -1.00 | -1.01 -0.99 | **<0.001** |  | -0.99 | -1.01 -0.99 | **<0.001** |
| **MBL T0** | -0.14 | -0.23 -0.04 | **0.005** |  | -0.01 | -0.01 0.01 | 0.532 |
| **Intrabony depth T0** | -0.13 | -0.21 -0.05 | **0.002** |  |  |  |  |
| **Defect depth T0** | 0.01 | 0.00 0.02 | **0.012** |  | 0.00 | 0.00 0.01 | 0.498 |
| **Recession T0** | 0.02 | -0.19 0.23 | 0.861 |  |  |  |  |
| **KM T0** | -0.02 | -0.13 0.10 | 0.786 |  |  |  |  |

**Supplementary table 6. Changes in MR by Group, patient´s profile and implant and defect characteristics:** Results from simple and multiple linear regression using GEE, non-adjusted and adjusted beta coefficients and 95% confidence intervals.

|  | **Simple** | | |  | **multiple** | | |
| --- | --- | --- | --- | --- | --- | --- | --- |
|  | **Beta** | **95% CI** | **p-value** |  | **Beta** | **95% CI** | **p-value** |
| **Group** |  |  |  |  |  |  |  |
| PLIP | 0 |  |  |  | 0 |  |  |
| FLIP | 1.02 | 0.39 1.66 | **0.002** |  | 0.92 | 0.26 1.58 | **0.006** |
| **Gender** |  |  |  |  |  |  |  |
| Male | 0 |  |  |  |  |  |  |
| Female | 0.15 | -0.56 0.86 | 0.676 |  |  |  |  |
| **Age** | 0.03 | -0.01 0.07 | **0.078** |  | -0.01 | -0.05 0.03 | 0.627 |
| **Smoking** |  |  | 0.522 |  |  |  |  |
| No | 0 |  |  |  |  |  |  |
| Former | 0.26 | -0.83 1.34 | 0.644 |  |  |  |  |
| Yes | -0.17 | -0.62 0.27 | 0.446 |  |  |  |  |
| **Prosthesis** |  |  |  |  |  |  |  |
| FPD | 0 |  |  |  |  |  |  |
| Rest | 0.39 | -0.40 1.17 | 0.334 |  |  |  |  |
| **Implant system** |  |  | 0.239 |  |  |  |  |
| Nobel Biocare | 0 |  |  |  |  |  |  |
| AstraTech | -0.81 | -1.99 0.36 | 0.176 |  |  |  |  |
| 3i | -0.31 | -0.74 0.11 | 0.151 |  |  |  |  |
| **Location** |  |  |  |  |  |  |  |
| PM | 0 |  |  |  | 0 |  |  |
| pm | -0.69 | -1.38 -0.01 | **0.047** |  | -0.97 | -1.58 -0.37 | **0.002** |
| **Defect type** |  |  | 0.121 |  |  |  |  |
| IB | 0 |  |  |  |  |  |  |
| IIIB | 0.75 | -0.04 1.54 | 0.063 |  |  |  |  |
| IIIC | 0.92 | -0.26 2.09 | 0.126 |  |  |  |  |
| **Defect extension** |  |  |  |  |  |  |  |
| AD | 0 |  |  |  |  |  |  |
| MO | 0.47 | -0.48 1.42 | 0.335 |  |  |  |  |
| **Early complications** |  |  |  |  |  |  |  |
| No | 0 |  |  |  |  |  |  |
| Yes | -0.46 | -1.01 0.09 | 0.105 |  |  |  |  |
| **PPD T0** | 0.03 | -0.22 0.28 | 0.828 |  |  |  |  |
| **mSBI T0** | -0.24 | -0.68 0.19 | 0.273 |  |  |  |  |
| **SUP T0** | 0.11 | -0.66 0.89 | 0.775 |  |  |  |  |
| **MBL T0** | 0.17 | -0.04 0.39 | 0.114 |  |  |  |  |
| **Intrabony depth T0** | 0.06 | -0.12 0.24 | 0.487 |  |  |  |  |
| **Defect depth T0** | 0.00 | -0.02 0.02 | 0.986 |  |  |  |  |
| **Recession T0** | -0.61 | -1.07 -0.15 | **0.010** |  | -0.39 | -0.73 -0.04 | **0.029** |
| **KM T0** | -0.21 | -0.48 0.06 | 0.119 |  |  |  |  |

**Supplementary table 7. Changes in KM by Group, patient´s profile and implant and defect characteristics:** Results from simple and multiple linear regression using GEE, non-adjusted and adjusted beta coefficients and 95% confidence intervals.

|  | **Simple** | | |  | **multiple** | | |
| --- | --- | --- | --- | --- | --- | --- | --- |
|  | **Beta** | **95% CI** | **p-value** |  | **Beta** | **95% CI** | **p-value** |
| **Group** |  |  |  |  |  |  |  |
| PLIP | 0 |  |  |  | 0 |  |  |
| FLIP | 0.27 | -0.62 1.16 | 0.555 |  | 0.31 | -0.40 1.02 | 0.385 |
| **Gender** |  |  |  |  |  |  |  |
| Male | 0 |  |  |  |  |  |  |
| Female | -0.51 | -1.69 0.67 | 0.396 |  |  |  |  |
| **Age** | 0.01 | -0.03 0.04 | 0.703 |  | -0.01 | -0.04 0.02 | 0.414 |
| **Smoking** |  |  | **0.005** |  |  |  | 0.264 |
| No | 0 |  |  |  | 0 |  |  |
| Former | 0.15 | -0.70 1.01 | 0.727 |  | -0.01 | -1.06 1.04 | 0.985 |
| Yes | -1.28 | -2.15 -0.40 | **0.004** |  | -0.95 | -2.11 0.21 | 0.109 |
| **Prosthesis** |  |  |  |  |  |  |  |
| FPD | 0 |  |  |  |  |  |  |
| Rest | 0.54 | -0.26 1.33 | 0.185 |  |  |  |  |
| **Implant system** |  |  | **<0.001** |  |  |  | 0.571 |
| Nobel Biocare | 0 |  |  |  | 0 |  |  |
| AstraTech | 0.78 | -1.55 3.12 | 0.512 |  | 0.79 | -1.52 3.09 | 0.503 |
| 3i | -1.22 | -1.63 -0.81 | **<0.001** |  | -0.39 | -2.07 1.30 | 0.653 |
| **Location** |  |  |  |  |  |  |  |
| PM | 0 |  |  |  |  |  |  |
| pm | 0.24 | -0.86 1.34 | 0.667 |  |  |  |  |
| **Defect type** |  |  | 0.599 |  |  |  |  |
| IB | 0 |  |  |  |  |  |  |
| IIIB | -0.64 | -1.89 0.61 | 0.315 |  |  |  |  |
| IIIC | -0.58 | -1.97 0.80 | 0.409 |  |  |  |  |
| **Defect extension** |  |  |  |  |  |  |  |
| AD | 0 |  |  |  |  |  |  |
| MO | -0.03 | -0.73 0.67 | 0.928 |  |  |  |  |
| **Early complications** |  |  |  |  |  |  |  |
| No | 0 |  |  |  | 0 |  |  |
| Yes | -0.89 | -1.61 -0.17 | **0.016** |  | -0.83 | -2.79 1.14 | 0.411 |
| **PPD T0** | 0.08 | -0.08 0.24 | 0.323 |  |  |  |  |
| **mSBI T0** | 0.04 | -0.43 0.52 | 0.862 |  |  |  |  |
| **SUP T0** | -0.22 | -0.94 0.51 | 0.555 |  |  |  |  |
| **MBL T0** | -0.13 | -0.46 0.20 | 0.439 |  |  |  |  |
| **Intrabony depth T0** | -0.12 | -0.40 0.16 | 0.404 |  |  |  |  |
| **Defect depth T0** | 0.01 | -0.02 0.04 | 0.421 |  |  |  |  |
| **Recession T0** | -0.26 | -0.68 0.16 | 0.218 |  |  |  |  |
| **KM T0** | -0.25 | -0.72 0.22 | 0.303 |  |  |  |  |

**Supplementary table 8. Changes in ID by Group, patient´s profile and implant and defect characteristics:** Results from simple and multiple linear regression using GEE, non-adjusted and adjusted beta coefficients and 95% confidence intervals.

|  | **Simple** | | |  | **multiple** | | |
| --- | --- | --- | --- | --- | --- | --- | --- |
|  | **Beta** | **95% CI** | **p-value** |  | **Beta** | **95% CI** | **p-value** |
| **Group** |  |  |  |  |  |  |  |
| PLIP | 0 |  |  |  | 0 |  |  |
| FLIP | -0.46 | -1.16 0.24 | 0.201 |  | -0.22 | -0.75 0.30 | 0.403 |
| **Gender** |  |  |  |  |  |  |  |
| Male | 0 |  |  |  | 0 |  |  |
| Female | 0.77 | -0.11 1.66 | **0.087** |  | 0.37 | -0.13 0.87 | 0.149 |
| **Age** | -0.04 | -0.06 -0.02 | **<0.001** |  | -0.01 | -0.05 0.02 | 0.510 |
| **Smoking** |  |  | 0.464 |  |  |  |  |
| No | 0 |  |  |  |  |  |  |
| Former | -0.75 | -1.96 0.46 | 0.225 |  |  |  |  |
| Yes | 0.11 | -1.17 1.38 | 0.869 |  |  |  |  |
| **Prosthesis** |  |  |  |  |  |  |  |
| FPD | 0 |  |  |  |  |  |  |
| Rest | 0.14 | -0.53 0.80 | 0.688 |  |  |  |  |
| **Implant system** |  |  | **<0.001** |  |  |  | 0.151 |
| Nobel Biocare | 0 |  |  |  | 0 |  |  |
| AstraTech | -1.08 | -2.56 0.39 | 0.150 |  | -0.40 | -1.47 0.68 | 0.467 |
| 3i | 0.99 | 0.50 1.49 | **<0.001** |  | 0.50 | -0.12 1.12 | 0.112 |
| **Location** |  |  |  |  |  |  |  |
| PM | 0 |  |  |  |  |  |  |
| pm | -0.25 | -1.03 0.53 | 0.531 |  |  |  |  |
| **Defect type** |  |  | 0.115 |  |  |  |  |
| IB | 0 |  |  |  |  |  |  |
| IIIB | 0.43 | -0.34 1.20 | 0.272 |  |  |  |  |
| IIIC | -0.46 | -1.34 0.42 | 0.303 |  |  |  |  |
| **Defect extension** |  |  |  |  |  |  |  |
| AD | 0 |  |  |  | 0 |  |  |
| MO | 0.63 | -0.01 1.27 | **0.053** |  | 0.49 | 0.03 0.95 | **0.035** |
| **Early complications** |  |  |  |  |  |  |  |
| No | 0 |  |  |  |  |  |  |
| Yes | 0.68 | -0.21 1.58 | 0.135 |  |  |  |  |
| **PPD T0** | 0.09 | -0.08 0.27 | 0.288 |  |  |  |  |
| **mSBI T0** | 0.23 | -0.28 0.74 | 0.375 |  |  |  |  |
| **SUP T0** | -0.56 | -1.20 0.08 | 0.186 |  |  |  |  |
| **MBL T0** | -0.38 | -0.62 -0.13 | **0.003** |  |  |  |  |
| **Intrabony depth T0** | -0.50 | -0.68 -0.32 | **<0.001** |  | -0.37 | -0.55 -0.20 | **<0.001** |
| **Defect depth T0** | 0.05 | 0.03 0.08 | **<0.001** |  |  |  |  |
| **Recession T0** | 0.11 | -0.25 0.47 | 0.554 |  |  |  |  |
| **KM T0** | 0.22 | 0.03 0.41 | **0.025** |  | 0.12 | -0.09 0.34 | 0.268 |

**Supplementary table 9. Changes in DA by Group, patient´s profile and implant and defect characteristics:** Results from simple and multiple linear regression using GEE, non-adjusted and adjusted beta coefficients and 95% confidence intervals.

|  | **Simple** | | |  | **multiple** | | |
| --- | --- | --- | --- | --- | --- | --- | --- |
|  | **Beta** | **95% CI** | **p-value** |  | **Beta** | **95% CI** | **p-value** |
| **Group** |  |  |  |  |  |  |  |
| PLIP | 0 |  |  |  | 0 |  |  |
| FLIP | 1.04 | -5.78 7.86 | 0.765 |  | -0.57 | -5.87 4.73 | 0.834 |
| **Gender** |  |  |  |  |  |  |  |
| Male | 0 |  |  |  |  |  |  |
| Female | -2.95 | -11.7 5.85 | 0.512 |  |  |  |  |
| **Age** | 0.52 | 0.28 0.75 | **<0.001** |  | 0.39 | -0.01 0.78 | 0.054 |
| **Smoking** |  |  | 0.287 |  |  |  |  |
| No | 0 |  |  |  |  |  |  |
| Former | 0.53 | -8.11 9.17 | 0.905 |  |  |  |  |
| Yes | -6.22 | -14.3 1.88 | 0.132 |  |  |  |  |
| **Prosthesis** |  |  |  |  |  |  |  |
| FPD | 0 |  |  |  |  |  |  |
| Rest | -1.33 | -9.03 6.38 | 0.736 |  |  |  |  |
| **Implant system** |  |  | **<0.001** |  |  |  | 0.994 |
| Nobel Biocare | 0 |  |  |  | 0 |  |  |
| AstraTech | 2.45 | -10.6 15.5 | 0.713 |  | 0.15 | -9.15 9.45 | 0.975 |
| 3i | -8.47 | -12.4 -4.57 | **<0.001** |  | -0.52 | -10.9 9.89 | 0.922 |
| **Location** |  |  |  |  |  |  |  |
| PM | 0 |  |  |  |  |  |  |
| pm | -1.40 | -8.81 6.02 | 0.712 |  |  |  |  |
| **Defect type** |  |  | 0.843 |  |  |  |  |
| IB | 0 |  |  |  |  |  |  |
| IIIB | 0.72 | -7.76 9.19 | 0.868 |  |  |  |  |
| IIIC | 3.15 | -7.93 14.2 | 0.578 |  |  |  |  |
| **Defect extension** |  |  |  |  |  |  |  |
| AD | 0 |  |  |  |  |  |  |
| MO | -4.62 | -11.0 1.82 | 0.159 |  |  |  |  |
| **Early complications** |  |  |  |  |  |  |  |
| No | 0 |  |  |  |  |  |  |
| Yes | -3.98 | -15.9 7.96 | 0.514 |  |  |  |  |
| **PPD T0** | -0.59 | -2.47 1.29 | 0.537 |  |  |  |  |
| **mSBI T0** | -1.16 | -5.59 3.26 | 0.606 |  |  |  |  |
| **SUP T0** | -1.07 | -8.78 6.65 | 0.787 |  |  |  |  |
| **MBL T0** | 1.22 | -1.05 3.49 | 0.292 |  |  |  |  |
| **Intrabony depth T0** | 2.04 | 0.25 3.83 | **0.026** |  |  |  |  |
| **Defect depth T0** | -0.46 | -0.62 -0.30 | **<0.001** |  | -0.37 | -0.56 -0.19 | **<0.001** |
| **Recession T0** | -1.20 | -5.62 3.22 | 0.595 |  |  |  |  |
| **KM T0** | -2.76 | -5.44 -0.07 | **0.044** |  | -0.46 | -3.33 2.41 | 0.751 |
